# Accurate Prediction of Immunogenic T-Cell Epitopes from Epitope Sequences Using the Genetic Algorithm-Based Ensemble Learning

**DOI:** 10.1371/journal.pone.0128194

**Published:** 2015-05-28

**Authors:** Wen Zhang, Yanqing Niu, Hua Zou, Longqiang Luo, Qianchao Liu, Weijian Wu

**Affiliations:** 1 School of Computer, Wuhan University, Wuhan, 430072, China; 2 Research Institute of Shenzhen, Wuhan University, Shenzhen, 518057, China; 3 School of Mathematics and Statistics, South-central University for Nationalities, Wuhan, 430074, China; 4 School of Mathematics and Statistics, Wuhan University, Wuhan, 430072, China; University of Catania, ITALY

## Abstract

**Background:**

T-cell epitopes play the important role in T-cell immune response, and they are critical components in the epitope-based vaccine design. Immunogenicity is the ability to trigger an immune response. The accurate prediction of immunogenic T-cell epitopes is significant for designing useful vaccines and understanding the immune system.

**Methods:**

In this paper, we attempt to differentiate immunogenic epitopes from non-immunogenic epitopes based on their primary structures. First of all, we explore a variety of sequence-derived features, and analyze their relationship with epitope immunogenicity. To effectively utilize various features, a genetic algorithm (GA)-based ensemble method is proposed to determine the optimal feature subset and develop the high-accuracy ensemble model. In the GA optimization, a chromosome is to represent a feature subset in the search space. For each feature subset, the selected features are utilized to construct the base predictors, and an ensemble model is developed by taking the average of outputs from base predictors. The objective of GA is to search for the optimal feature subset, which leads to the ensemble model with the best cross validation AUC (area under ROC curve) on the training set.

**Results:**

Two datasets named ‘IMMA2’ and ‘PAAQD’ are adopted as the benchmark datasets. Compared with the state-of-the-art methods POPI, POPISK, PAAQD and our previous method, the GA-based ensemble method produces much better performances, achieving the AUC score of 0.846 on IMMA2 dataset and the AUC score of 0.829 on PAAQD dataset. The statistical analysis demonstrates the performance improvements of GA-based ensemble method are statistically significant.

**Conclusions:**

The proposed method is a promising tool for predicting the immunogenic epitopes. The source codes and datasets are available in S1 File.

## Background

A vaccine is a biological preparation, which stimulates the production of antibodies to induce immunity to a particular disease. There are different types of vaccines. The epitope-based vaccine is a new kind of vaccine that recently attracts the wide interests. Critical components in manufacturing epitope-based vaccines are epitopes, which are designed to trigger the immune responses of T-cells or B-cells.

The intracellular antigen-processing pathway for T-cell immune responses is a complex procedure. At first, antigens are cleaved into short peptides, and some peptides are transported into the endoplasmic reticulum (ER) by the antigen presenting proteins. Then, some peptides will bind to major histocompatibility complex (MHC) molecules and form the MHC-peptide complexes. Finally, the complexes are presented on the cell surface to induce the immune response.

T-cell epitopes are defined as the antigen segments that bind to major histocompatibility molecules. The major histocompatibility complex (MHC) is the cell surface molecules in vertebrates that are encoded by a specified gene family. The MHC molecules are of two sorts: MHC-I and MHC-II. MHC-I molecules usually present epitopes of 9 amino acids, whereas epitopes binding to MHC-II may consist of 12–25 amino acids. In the study, we discuss the MHC-I restricted T-cell epitopes, which are also known as ‘CTL epitopes’. In the following context, T-cell epitopes refer to the CTL epitopes.

Wet methods that recognize T-cell epitopes are laborious and time-consuming, while computational methods are capable of reducing time and saving resources for the development of epitope-based vaccines. In recent years, the increasing coverage of experimental data and the development of intelligent techniques lead to the growth of computational methods. These prediction methods are designed for different stages of intracellular antigen-processing pathway, i.e. antigen cleavage [1–3], peptide transport [4–5] and MHC binding [6–17]. In addition, some computational methods that integrate multiple pathway steps were further developed [18–21].

In the design of vaccines, the primary consideration is to reduce risk and retain capability of inducing immune responses. Immunogenicity is the ability to trigger immune responses. Some studies showed that epitopes have the potential of activating the immune response but have not always been immunogenic. In other words, some epitopes can activate the immune response, and the others cannot. Because epitopes are classified into immunogenic epitopes and non-immunogenic epitopes, the work of predicting immunogenic epitopes is challenging and valuable.

A lot of studies [22–24] have been focused on crystal structures of the MHC-peptide complexes, but few useful conclusions were drawn because of the limited number of complex structures. Considering the fact that there are much more epitope sequences than epitope structures in databases, researchers make efforts to develop machine-learning prediction models based on epitope sequences. As far as we know, four machine-learning methods were proposed to predict the immunogenic epitopes. POPI [25] is the first method for immunogenic epitope prediction. This method selected 23 informative amino acid propensities from AAIndex database, and then utilized support vector machine classifier (SVM) to construct the prediction models. POPISK [26] is a SVM-based method using the weighted degree string kernel. PAAQD [27] adopted two novel sequence-derived features (the amino acid pairwise contact potentials and the quantum topological molecular similarity), and the SVM-based predictor was constructed. In the previous work [28], we proposed the average scoring ensemble method by combining seven sequence-derived features. Specifically, individual feature-based classifiers were used as base predictors, and the ensemble model takes the average of the outputs from base predictors for prediction.

To the best of our knowledge, there are some key issues for developing high-accuracy models. Firstly, the accuracy of models is highly dependent on the diversity of features. In order to achieve high-accuracy models, we should consider as many sequence-derived features as possible. Secondly, how to effectively combine various features to build high-accuracy model is very challenging. Considering the redundant information between features, the model using all features does not necessarily lead to the best result than a model using a subset of features. Therefore, we need to solve the problem that which features should be selected for modeling.

In this paper, we address above issues to make accurate predictions for the immunogenic epitopes. In order to obtain the diversity of features, we consider 18 sequence-derived features, which describe the sequential and structural characteristics of epitope sequences. Then, a genetic algorithm (GA)-based ensemble method is proposed to simultaneously determine the optimal feature subset for ensemble learning and develop the high-accuracy ensemble model. In the GA optimization, a chromosome is to represent a subset of features in the search space. For each chromosome, the selected features are adopted to construct base predictors, and then an ensemble model is developed by taking the average of outputs from base predictors. The fitness score of a chromosome is the 10-fold cross validation AUC (area under ROC curve) of the corresponding ensemble model on the training set. After an initial population is generated, the population is updated by three operators: selection, mutation and crossover. The objective of GA is to search for the optimal feature subset in the large search space, which leads to the ensemble model with best AUC score. Compared with the state-of-the-art methods, the proposed GA-based ensemble method yields much better performances on benchmark datasets, indicating it is a promising tool for the immunogenic epitope prediction.

## Methods

### 2.1. Datasets

To the best of our knowledge, there are several immune databases such as SYFPEITHI [29] and IEDB [30], which contain immunogenic epitopes. SYFPEITHI is a database with more than 7000 MHC-binding peptide sequences. In addition, SYFPEITHI can provide the retrieval of sequences and the epitope prediction. The Immune Epitope Database (IEDB) contains a catalog of experimentally characterized T-cell epitopes. Moreover, the epitope structures, source antigens and epitope-derived organisms are annotated. We retrieved MHC-binding peptides with immunogenicity information from above databases, and compiled them in terms of MHC alleles. However, after removing duplicate entries and high homology sequences, only the MHC allele HLA-A2 contains enough epitope sequences (more than 100 sequences) for the study on the immunogenic epitopes. Therefore, we directly adopt the datasets used in the previous studies [25–28].

We assess the performances of the proposed method on two publicly available datasets. One dataset has been used in the development of POPISK [26]. It consists of 558 HLA-A2 restricted immunogenic epitopes and 527 non-immunogenic epitopes. This dataset named as 'IMMA2 dataset' is available at http://iclab.life.nctu.edu.tw/POPISK/download.php. The other dataset has been applied to PAAQD [27], and we name it 'PAAQD dataset'. This dataset contains 278 HLA-A2 restricted immunogenic epitopes and 101 non-immunogenic epitopes, available at http://pirun.ku.ac.th/~fsciiok/PAAQD.rar. Two datasets have no duplicate sequences, and IMMA2 dataset contains much more epitope sequences than PAAQD dataset. We use IMMA2 dataset to analyze and evaluate the features, and compare the proposed methods with state-of-the-art methods by using two benchmark datasets.

### 2.2 Sequence-Derived Features

Our work is to differentiate immunogenic epitopes from non-immunogenic epitopes. The first step for the immunogenic epitope prediction is to represent the protein sequences with certain encoding scheme. In this work, we extract 18 protein sequence-derived features that are commonly used to predict protein functions, with the aim of obtaining diversity. Seven out of 18 features have been utilized for the immunogenic epitope prediction, while the rest are taken into account for the first time.

Physicochemical propensities: 11 physicochemical propensities from AAindex database [31] (AAindex IDs: MEEJ800102, WOLS870102, CASG920101, NAKH900110, FASG760105, FAUJ880105, CHAM830107, QIAN880127, RACS820108, DIGM050101 and TANS770109) were proved to be useful for the immunogenic epitope prediction [26].

Amino acid pairwise contact potentials (AAPPs) and quantum topological molecular similarity (QTMS): AAPPs describes the potentials between peptide amino acids and MHC amino acids. QTMS includes both physical and topological properties of peptides. Saethang et al. [27] used AAPPs and QTMS descriptors to represent epitopes in the immunogenic epitope prediction. Details about AAPPs and QTMS are available in [27].

Amino acid composition (AAC): the amino acid composition denotes the percentage of each of 20 amino acids in a sequence [32], and the AAC of a sequence is a 20-dimensional numeric vector.

Amino acid pair profile: the amino acid pair profile [33] of a sequence is a 400-dimensional number vector, and each dimension is the percentage of an amino acid pair pattern.

Sparse profile: by encoding each amino acid type as a 20-bit vector with 19 bits set to zero and one bit set to one, the sparse profile [34] of a sequence is obtained by merging the bit vectors for its amino acids.

Pairwise similarity profile: the pairwise similarity profile [35] of a sequence is represented by a numerical vector, which consists of the Smith-Waterman pairwise similarity scores between it and all sequences in the dataset.

Composition (CTDC), transition (CTDT), and distribution (CTDD): by dividing 20 amino acids into three groups in terms of different amino acid attributes, CTDC, CTDT, and CTDD [36] are respectively used to describe the global percent composition of each group in a sequence, global percent composition of group changes along the entire length of the sequence, and distribution pattern of each group along the sequence.

Autocorrelation: autocorrelation descriptors [37] are defined based on the distribution of amino acid properties along sequences. There are three kinds of autocorrelation, namely Normalized Moreau-Broto autocorrelation, Geary autocorrelation and Moran autocorrelation.

Quasi-sequence-order (QSO): QSO [38] is to incorporate physicochemical distance matrix between each pair of the 20 amino acids, and it reflects the sequence order coupling number based on the physicochemical distance.

Pseudo amino acid Composition (PseAA): PseAA [39] is proposed to avoid losing the sequence-order information. PseAA of a sequence consists of two components. The first component represents amino acid composition while the other component reflects the sequence-order information.

Amphiphilic Pseudo Amino Acid Composition (AmPseAA): AmPseAA [40] is an extension of Pseudo-Amino Acid Composition, and it integrates a set of correlation factors that reflect different hydrophobicity and hydrophilicity distribution patterns along a protein chain.

Secondary structures (SS) and relative accessible surface areas (RASA): secondary structures and relative accessible surface areas of residues in a sequence are calculated by the SABLE program [41]. The predicted SS is denoted as H, E or C (helix, sheet, coil). We use (1, 0, 0), (0, 1, 0) and (0, 0, 1) to represent the residue that belongs to three types of secondary structures, respectively. The predicted RASA of a residue is a real value between 0 and 100, representing the percentage of exposed area of the residue over its full area.

These features are summarized in Table 1.

**Table 1:** Details about sequence-derived features.

Index	Feature	Dimension	Parameters	Annotation
F1	Physicochemical propensities	99	No parameter	used in [26,28]
F2	Amino acid composition (AAC)	20	No parameter	used in [28]
F3	Amino acid pair profile	400	No parameter	used in [28]
F4	Sparse profile	20	No parameter	used in [28]
F5	Pairwise similarity profile	*n*	No parameter	used in [28]
F6	AAPPs	360	No parameter	used in [27,28]
F7	QTMS	189	No parameter	used in [27,28]
F8	Amino acid composition (CTDC)	21	No parameter	New feature
F9	Amino acid Transition (CTDT)	21	No parameter	New feature
F10	Amino acid Distribution (CTDD)	105	No parameter	New feature
F11	Moran autocorrelation	8×λ	λ, the lag of the autocorrelation	New feature
F12	Geary autocorrelation	8×λ	λ, the lag of the autocorrelation	New feature
F13	MoreauBroto autocorrelation	8×λ	λ, the lag of the autocorrelation	New feature
F14	Quasi-sequence-order (QSO)	40+2×λ	*λ*, the number of sequence order factors	New feature
F15	Pseudo Amino Acid Composition (PseAA)	20+λ	*λ*, the number of sequence order factors	New feature
F16	Amphiphilic Pseudo Amino Acid Composition (AmPseAA)	20+2×λ	*λ*, the number of sequence order factors	New feature
F17	Predicted relative accessible surface areas (RASA)	9	No parameter	New feature
F18	Predicted secondary structure (SS)	27	No parameter	New feature

* *n* is the number of sequences in the dataset, 0<λ< *L(sequence length)*, the new feature means that the features were not used in the immunogenic epitope prediction

### 2.3. The GA-Based Ensemble Method

Combining informative features helps to make high-accuracy prediction, because various features describe different characteristics of immunogenic epitopes. However, redundant information or unwanted noise is the main concern for feature combination.

In machine learning, the work that combines various features is also known as feature fusion, whose purpose is to exploit features and remove the redundant information. Merging various feature vectors is a simple and widely used feature fusion approach, whereas the ensemble learning is a sophisticated technique. Recently, ensemble learning attracts more and more interests in bioinformatics for their unique advantages in dealing with high-dimensional and complicated data [42–43].

In this paper, we design a genetic algorithm (GA)-based ensemble method for the immunogenic epitope prediction. There are two crucial components for designing the ensemble system, including base predictors and the rule for integrating base predictors. To develop the base predictors, the training sequences can be respectively encoded into different sets of feature vectors by using different features, and the individual feature-based classifiers are built based on the different sets of feature vectors. Here, the random forest (RF) [44] is adopted as the basic classification engine. Then, the individual feature-based RF classifiers are used as the base predictors. Further, we adopt the average scoring ensemble rule to integrate base predictors, and it takes the average of outputs from base predictors to make predictions.

Considering that each feature corresponds to a base predictor, there are 18 candidate base predictors for the ensemble learning. As discussed above, the ensemble based on all features does not always lead to the best result than an ensemble model based on a subset of features. The key to our ensemble method is to select optimal features for base predictors, which can lead to the best ensemble model. There are 262144 (2^18^) possible feature subsets for 18 features, and it is difficult to make the exhaustive search in such large search space. The genetic algorithm (GA) is a search approach that mimics the process of natural selection. GA can effectively search the interesting space and easily solve complex problems without requiring the prior knowledge about the space and the problem. Here, we adopt GA to simultaneously determine the optimal feature subset and build the high-accuracy ensemble model.

**Fig 1:**
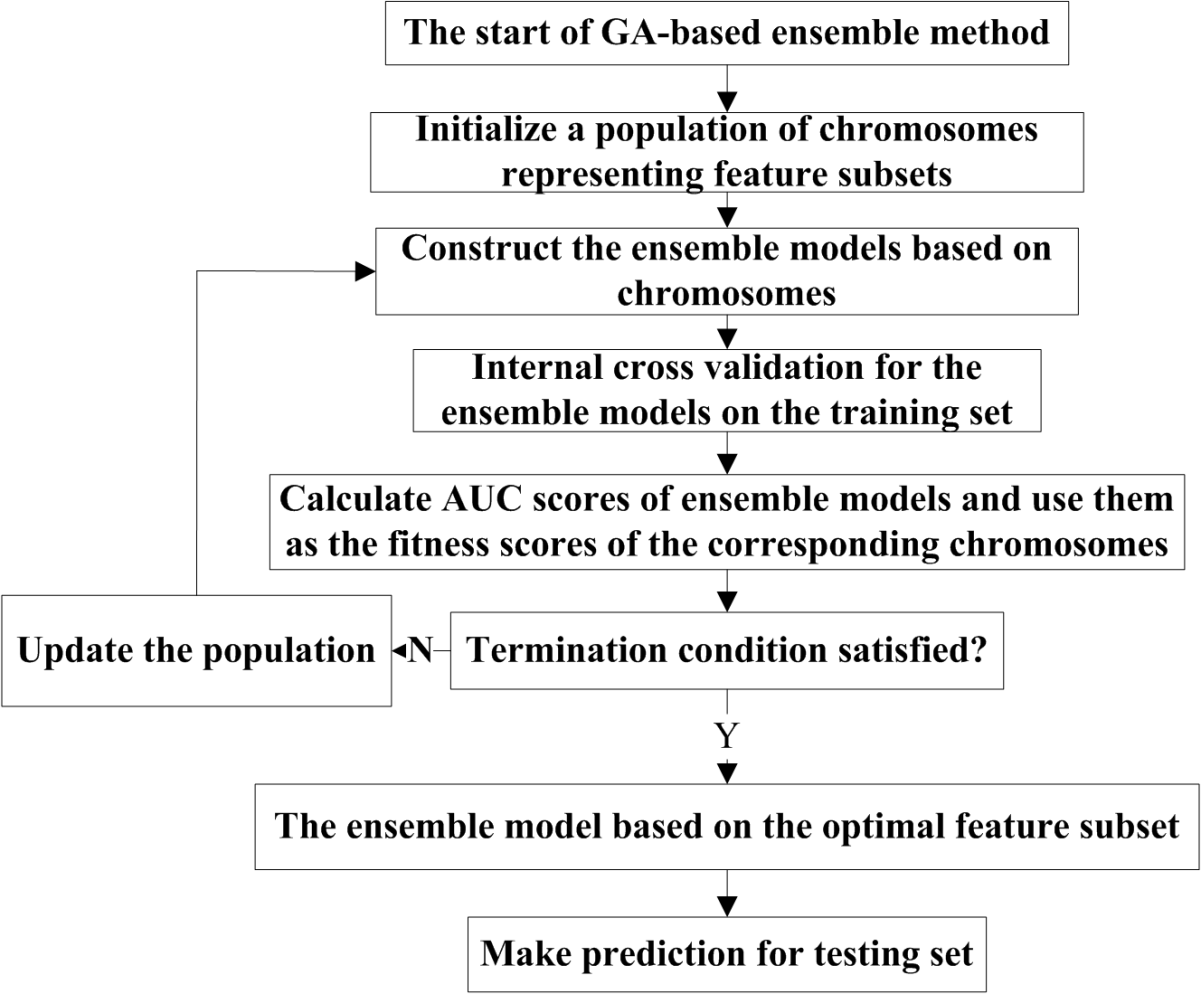
The flowchart of GA-based ensemble method.

Fig 1 demonstrates the flowchart of the GA-based ensemble method. The data is split into training set and testing set. The optimal feature subset and the corresponding ensemble model are achieved on the training set, and then the model is applied to the testing set.

The first step of GA is to encode the possible solutions as the chromosomes. For 18 features, a subset of features is naturally represented by a 18-dimensional binary vector, denoted as V = {v_1_, v_2_, …, v_18_}. The binary value 0 or 1 for each dimension indicates the absence or presence of the corresponding feature. A good fitness function is essential to assessing the performance of each chromosome. For each chromosome, base predictors are built by using selected features in the chromosome, and then an average scoring ensemble model is developed. An internal 5-fold cross validation is implemented for the ensemble model on the training set, and AUC of the ensemble model is taken as the fitness score of the chromosome. After randomly generating an initial population, the population is updated by three operators: selection, crossover and mutation, according to the fitness scores of chromosomes. GA optimization is to search for the chromosome that maximizes the AUC score.

Here, we use the Matlab GA toolbox and Matlab random forest toolbox (http://code.google.com/p/randomforest-matlab/) to implement the GA optimization and random forest classifiers. The default parameters are adopted for random forests.

## Results and Discussion

### 3.1. Performance Evaluation Metrics

The proposed methods are constructed on the benchmark datasets, and evaluated by the 10-fold cross-validation (10-CV). In the 10-CV, a dataset is randomly split into 10 subsets with equal size. In each fold, one subset is used as the testing data and the rest is treated as training data. The prediction model is trained on the training data, and then it is applied to the testing data. This procedure is repeated until every subset is ever used for testing.

Here, we adopt several metrics to measure the performances of prediction models, namely sensitivity (*SN*), specificity (*SP*), accuracy (*ACC*), Matthew’s correlation coefficient (*MCC*) and the area under the ROC curve (*AUC*). These metrics are defined as follows.

$$SN=\frac{TP}{TP+FN}$$

$$SP=\frac{TN}{TN+FP}$$

$$ACC=\frac{TP+TN}{TP+TN+FP+FN}$$

$$MCC=\frac{TP\times TN-FP\times FN}{\sqrt{\left(TP+FN)\times (TP+FP)\times (TN+FP)\times (TN+FN\right)}}$$

where *TP*, *TN*, *FP* and *FN* are the number of true positives, the number of true negatives, the number of false positives and the number of false negatives. The ROC curve is plotted by using the false positive rate (1-specificity) against the true positive rate (sensitivity) for different cut-off thresholds. AUC evaluates the performances regardless of any threshold, and it is adopted as the primary metric in this study.

### 3.2. Evaluation of Various Features

Before constructing prediction models, we investigate the sequence-derived features and analyze their capabilities of discriminating immunogenic epitopes from non-immunogenic epitopes.

As shown in Table 1, the dimensions of six sequence-derived features (e.g., indexed from F11 to F16) are related to a parameter *λ*, while the rest of features have fixed dimensions. For the features indexed from F11 to F13, the parameter *λ* denotes the lag of the autocorrelation; while for the features indexed from F14 to F16, the parameter *λ* denotes the additional length or dimensions of sequence order factors. Note that *λ* is an integer and 1< = *λ*< = *L*-1, where *L* is the sequence length (e.g., *L* = 9 in our work). To test the impact of *λ* on six features, the prediction models are constructed based on these features, for which different values of *λ* ranging from 1 to 8 are adopted. We conduct 20 independent runs of 10-CV for each model to avoid the bias of the data split, and the mean scores demonstrated in Table 2. It is observed that six features are to some extent influenced by the parameter *λ*. Take the Geary autocorrelation as an example, the variation of *λ* can cause a difference of approximately 7% AUC. The optimal values of *λ* that yield best results are used for six features in the following context.

**Table 2:** The average AUC scores of individual feature-based models using different values for λ, evaluated on IMMA2 by 20 independent runs of the 10-CV.

Parameter λ	1	2	3	4	5	6	7	8	Optimal value
Moran autocorrelation	0.567	0.585	0.592	0.605	0.604	0.617	**0.633**	0.631	7
Geary autocorrelation	0.571	0.580	0.588	0.608	0.609	0.616	**0.640**	0.636	7
MoreauBroto autocorrelation	0.655	0.663	0.659	0.674	0.679	0.680	**0.684**	0.684	7
Quasi-sequence-order	0.704	0.708	0.708	0.712	0.716	0.721	0.718	**0.723**	8
Pseudo Amino Acid Composition	0.704	0.699	0.705	0.701	0.708	0.709	0.705	**0.713**	8
Amphiphilic Pseudo Amino Acid Composition	0.691	**0.719**	0.712	0.715	0.708	0.704	0.707	0.707	2

Further, 18 sequence-derived features are evaluated on the IMMA2 datasets. More specifically, prediction models are respectively constructed by encoding epitope sequences with various features, and the AUC scores of individual feature-based models help to rank features. As shown in Table 3, AUC scores of individual feature-based models range from 0.58 to 0.78. Accordingly, the performances of these features are categorized into three groups: 'Exceptional' (>0.7),'Good' (< = 0.7 and >0.6) and ‘Poor’ (< = 0.6). In general, there are 10 ‘exceptional’ features, 7 ‘good’ features and 1 ‘poor’ feature. The predicted relative accessible surface area (RASA) yields the best results, indicating the structural information is the most important clue that recognizes the immunogenic epitopes.

**Table 3:** The average performances of different individual feature-based models, evaluated on IMMA2 by 20 independent runs of the 10-CV.

#	Feature	SN	SP	ACC	MCC	AUC
F1	Physicochemical propensities	0.507	0.847	0.672	0.377	0.738
F2	Amino acid composition (AAC)	0.574	0.701	0.636	0.289	0.693
F3	Amino acid pair profile	0.541	0.793	0.664	0.348	0.718
F4	Sparse profile	0.523	0.811	0.663	0.352	0.725
F5	Pairwise similarity profile	0.692	0.680	0.687	0.375	0.741
F6	AAPPs	0.550	0.813	0.678	0.382	0.747
F7	QTMS	0.507	0.825	0.662	0.354	0.732
F8	Amino acid composition (CTDC)	0.730	0.523	0.629	0.262	0.667
F9	Amino acid Transition (CTDT)	0.512	0.742	0.624	0.266	0.671
F10	Amino acid Distribution (CTDD)	0.592	0.743	0.666	0.340	0.720
F11	Moran autocorrelation	0.337	0.868	0.595	0.246	0.633
F12	Geary autocorrelation	0.333	0.861	0.589	0.238	0.640
F13	MoreauBroto autocorrelation	0.411	0.847	0.623	0.293	0.684
F14	Quasi-sequence-order (QSO)	0.626	0.724	0.674	0.352	0.723
F15	Pseudo Amino Acid Composition (PseAA)	0.661	0.657	0.659	0.325	0.713
F16	Amphiphilic Pseudo Amino Acid Composition (AmPseAA)	0.664	0.646	0.655	0.325	0.719
F17	Predicted relative accessible surface areas (RASA)	0.643	0.781	0.710	0.430	0.783
F18	Predicted secondary structure (SS)	0.917	0.295	0.615	0.273	0.585

### 3.3. Analysis on Redundant Information between Features

The sequence-derived features display different capability of predicting immunogenic epitopes. However, the redundant information or correlation between features may have the negative impact on combining these features.

To investigate the correlations between features, the individual feature-based models are evaluated on IMMA2 dataset by 20 independent runs of 10-CV, and Pearson correlation coefficients are calculated between the AUC scores of any two individual feature-based models. In statistics, Pearson correlation coefficient is a widely used measure of the linear dependence between two variables. The correlation coefficient is a real value between -1 and 1, where 1 is totally positive correlation, 0 is no correlation, and −1 is totally negative correlation. Here, we take the absolute values of correlation coefficients to measure the redundant information or correlations between features. The results in Table 4 show some features are highly correlated, such as F6 and F1, F6 and F7, indicating the difficulty of utilizing these features.

**Table 4:** The absolute values of correlation coefficients of AUC scores yielded by individual feature-based models

	F1	F2	F3	F4	F5	F6	F7	F8	F9	F10	F11	F12	F13	F14	F15	F16	F17	F18
**F1**	1.00	0.57	0.00	0.50	0.18	0.51	0.67	0.07	0.51	0.41	0.05	0.19	0.05	0.38	0.49	0.39	0.19	0.34
**F2**	0.57	1.00	0.18	0.69	0.52	0.42	0.56	0.11	0.29	0.40	0.07	0.27	0.02	0.60	0.56	0.38	0.27	0.02
**F3**	0.00	0.18	1.00	0.08	0.02	0.02	0.30	0.22	0.15	0.09	0.20	0.17	0.19	0.05	0.02	0.26	0.13	0.04
**F4**	0.50	0.69	0.08	1.00	0.63	0.47	0.82	0.03	0.53	0.49	0.17	0.01	0.02	0.41	0.45	0.15	0.27	0.13
**F5**	0.18	0.52	0.02	0.63	1.00	0.51	0.50	0.01	0.42	0.03	0.28	0.19	0.37	0.33	0.13	0.22	0.01	0.12
**F6**	0.51	0.42	0.02	0.47	0.51	1.00	0.68	0.10	0.45	0.19	0.04	0.08	0.28	0.11	0.09	0.29	0.14	0.10
**F7**	0.67	0.56	0.30	0.82	0.50	0.68	1.00	0.05	0.53	0.44	0.26	0.12	0.01	0.39	0.40	0.12	0.16	0.24
**F8**	0.07	0.11	0.22	0.03	0.01	0.10	0.05	1.00	0.07	0.07	0.06	0.67	0.10	0.14	0.38	0.10	0.02	0.21
**F9**	0.51	0.29	0.15	0.53	0.42	0.45	0.53	0.07	1.00	0.39	0.03	0.12	0.41	0.21	0.33	0.20	0.08	0.19
**F10**	0.41	0.40	0.09	0.49	0.03	0.19	0.44	0.07	0.39	1.00	0.12	0.05	0.03	0.56	0.65	0.17	0.33	0.02
**F11**	0.05	0.07	0.20	0.17	0.28	0.04	0.26	0.06	0.03	0.12	1.00	0.17	0.45	0.19	0.10	0.30	0.00	0.40
**F12**	0.19	0.27	0.17	0.01	0.19	0.08	0.12	0.67	0.12	0.05	0.17	1.00	0.29	0.26	0.23	0.25	0.18	0.23
**F13**	0.05	0.02	0.19	0.02	0.37	0.28	0.01	0.10	0.41	0.03	0.45	0.29	1.00	0.18	0.02	0.28	0.31	0.22
**F14**	0.38	0.60	0.05	0.41	0.33	0.11	0.39	0.14	0.21	0.56	0.19	0.26	0.18	1.00	0.80	0.48	0.13	0.08
**F15**	0.49	0.56	0.02	0.45	0.13	0.09	0.40	0.38	0.33	0.65	0.10	0.23	0.02	0.80	1.00	0.50	0.15	0.02
**F16**	0.39	0.38	0.26	0.15	0.22	0.29	0.12	0.10	0.20	0.17	0.30	0.25	0.28	0.48	0.50	1.00	0.25	0.05
**F17**	0.19	0.27	0.13	0.27	0.01	0.14	0.16	0.02	0.08	0.33	0.00	0.18	0.31	0.13	0.15	0.25	1.00	0.35
**F18**	0.34	0.02	0.04	0.13	0.12	0.10	0.24	0.21	0.19	0.02	0.40	0.23	0.22	0.08	0.02	0.05	0.35	1.00

In order to test the negative impact of redundant information, we directly combine the features by merging feature vectors. Here, seven feature combinations are generated by adding one feature once, in descending order of their AUC scores presented in Table 3. For simplicity, we do not test all feature combination, but it would not influence our analysis. For each feature combination, different groups of feature vectors are merged, and then the prediction model is constructed. According to Table 5, the model based on AAPPs and RASA yields the best results. Therefore, we can conclude that more features do not necessarily lead to better performance.

**Table 5:** The average performances of models merging different feature vectors, evaluated by 20 independent runs of the 10-CV.

#	Feature	SN	SP	ACC	MCC	AUC
Combination 1	F17+F6	0.692	0.758	0.724	0.455	0.799
Combination 2	F17+F6+F5	0.658	0.767	0.711	0.430	0.783
Combination 3	F17+F6+F5+F1	0.663	0.763	0.712	0.430	0.782
Combination 4	F17+F6+F5+F1+F7	0.652	0.774	0.711	0.431	0.783
Combination 5	F17+F6+F5+F1+F7+F4	0.639	0.782	0.708	0.429	0.782
Combination 6	F17+F6+F5+F1+F7+F4+F14	0.631	0.793	0.710	0.432	0.782
Combination 7	F17+F6+F5+F1+F7+F4+F14+F10	0.653	0.770	0.710	0.428	0.781

### 3.4. Performances of GA-based Ensemble Method

Given a variety of sequence-derived features, selecting features that are used for base predictors is the key to building high-accuracy ensemble model. Therefore, we apply GA to determine the optimal subset and develop the ensemble model.

The configurations for GA are described as follows. The initial population has 100 chromosomes. In the update of population, the elitist strategy is used for the selection operator, and default parameters in the Matlab GA toolbox are adopted for the mutation probability and crossover probability. The population update may terminate when the change of best fitness scores is less than the default value of 1E-6 or the max generation number of 100 is reached.

The results of GA-based ensemble method on IMMA2 dataset and PAAQD dataset are given in Table 6. The GA-based ensemble method produces the AUC score of 0.846 on IMMA2 dataset and AUC score of 0.829 on PAAQD dataset. We compare the ensemble models with the individual feature-based models, according to Table 2 and Table 6. Clearly, the GA-based ensemble method produces much better results, indicating this ensemble approach can effectively combine various features to enhance performances.

**Table 6:** The average performances of GA-based ensemble method on benchmark datasets, evaluated by 20 runs of 10-CV.

Dataset	SN	SP	ACC	MCC	AUC
IMMA2	0.715	0.812	0.762	0.534	0.846
PAAQD	0.919	0.534	0.817	0.509	0.829

Further, we analyze the optimal subsets that are identified by GA. In each fold of 10-CV, the optimal feature subset is automatically determined by internal cross validation on the training set. There are 200 optimal feature subsets (20×10) for 20 independent runs of 10-CV. Here, we count the frequencies of features appearing in the optimal subsets, and then we may have some useful observations from the results in Table 7. Firstly, these optimal feature subsets are different, because of the different training data. Secondly, the optimal feature subsets do not consist entirely of the highly ranked features. Instead, an optimal subset may include some ‘poor’ features. For example, the secondary structure (F18) is ever included in 18 optimal subsets. Thirdly, size of optimal subsets ranges from 2 features to 5 features. In conclusion, the optimal feature subset for the ensemble model depends on the training data, and identifying the optimal feature subset is necessary for building high-accuracy models.

**Table 7:** The frequencies of features in the optimal feature subsets.

Index	F1	F2	F3	F4	F5	F6	F7	F8	F9	F10	F11	F12	F13	F14	F15	F16	F17	F18
Frequencies	4	0	17	3	145	77	97	0	1	30	0	7	0	2	1	24	200	11

### 3.5. Comparison with Benchmark Methods

As far as we know, four computational methods (POPI [25], POPISK [26], PAAQD [27] and our previous method [28]) were proposed to predict immunogenic epitopes. Therefore, we adopt these methods as benchmark methods for comparison.

In this paper, we compare the proposed GA-based ensemble method with four state-of-the-art methods on IMMA2 dataset and PAAQD dataset. The models are evaluated by 10-fold cross validation. In addition, the significance of improvements is tested by statistical techniques.

First of all, we have to obtain the 10-CV performances of different methods. The source codes for POPI and POPISK are not publicly available, and it is difficult to correctly replicate these methods because details are ambiguous. For the fair comparison, we have to directly adopt their results, which were reported in [25, 26]. Although 20 independent runs of 10-CV were ever implemented for POPI and POPISK on IMMA2 dataset, only the average scores are available in the publications, and some scores such as sensitivity and specificity are not provided. The standalone R package of PAAQD can be downloaded at http://pirun.ku.ac.th/~fsciiok/PAAQD.rar, and we could replicate PAAQD to obtain the results on IMMA2 dataset and PAAQD dataset. Therefore, we conduct 20 independent runs of 10-CV for PAAQD, our previous method and GA-based ensemble method to obtain the scores.

As shown in Table 8, POPI [25], POPISK [26], PAAQD [27] and our previous method [28] produce the AUC scores of 0.64, 0.74, 0.747 and 0.766 on the IMMA2 dataset, while the GA-based ensemble method produces the AUC score of 0.846. The GA-based ensemble method also yields much better performances than PAAQD [27] and our previous method [28] on the PAAQD dataset, enhancing the AUC scores from 0.773 to 0.829.

**Table 8:** The average performances of different models evaluated by 20 independent runs of 10-CV.

Dataset	Method	SN	SP	ACC	MCC	AUC
IMMA2	POPI	N.A.	N.A.	0.60	0.19	0.64
	POPISK	N.A.	N.A.	0.68	0.37	0.74
	PAAQD	0.523	0.832	0.673	0.379	0.747
	Our previous method	0.573	0.818	0.692	0.406	0.766
	GA-based ensemble method	**0.715**	**0.812**	**0.762**	**0.534**	**0.846**
PAAQD	PAAQD	0.508	0.898	0.612	0.373	0.749
	Our previous method	0.548	0.902	0.642	0.403	0.773
	GA-based ensemble method	**0.919**	**0.534**	**0.817**	**0.509**	**0.829**

*N.A. means data not available.

Although the GA-based ensemble method yields much better performances than benchmark methods, we further adopt the statistical technique to test the significance of performance improvements. Since we implemented 20 independent runs of 10-CV for PAAQD method, our previous method and GA-based ensemble method, we obtain 20 samples for each method by considering the AUC score in a run as a sample. Therefore, the paired t-test is used to compare GA-based ensemble method with PAAQD and our previous method. As discussed above, only the average scores of 20 runs were given for POPI and POPISK [25, 26]. Following the work of PAAQD [27], we have to take the one-sample t-test to test 20 samples of the GA-based ensemble method against the average AUC scores of POPI and POPISK. The results in Table 9 indicate that the improvements of the GA-based ensemble method over benchmark methods are statistically significant (*P*<0.05 in terms of AUC scores).

**Table 9:** The statistics of improvements over benchmark methods (significance level 0.05).

Dataset	Method	POPI	POPISK	PAAQD	Our previous method
IMMA2	GA-based ensemble method	1.9E-16	3.0E-11	4.0E-22	1.3E-20
PAAQD	GA-based ensemble method	N.A.	N.A.	3.3E-14	3.5E-12

*N.A. means data not available.

There are some reasons for the superior performances of the GA-based ensemble method. Firstly, we consider a great number of sequence-derived features, which can guarantee the diversity for ensemble learning. Secondly, the GA-based ensemble method automatically determines the optimal feature subsets for base predictors, for the purpose of incorporating the useful information and reducing the information redundancy. Thirdly, the ensemble model equally treats base predictors, and the ensemble rule does not use any prior knowledge that may affect the performances.

## Conclusion

The prediction of T-cell immunogenic epitopes is a crucial task in the immunoinformatics, which can facilitate the epitope-based vaccine design. In this paper, we explore a wide variety of sequence-derived features that make differences between immunogenic epitopes and non-immunogenic epitopes. Then, we propose the GA-based ensemble method to automatically select the optimal feature subset for base predictors, and develop the ensemble model for the immunogenic epitope prediction. When compared with the state-of-the-art methods POPI, POPISK, PAAQD and our previous method, the GA-based ensemble method produces much better performances on the benchmark datasets, and the t-test analysis demonstrates that the performance improvements of the GA-based ensemble method are statistically significant. Nevertheless, there are some remaining spaces for improving our work. Currently, only the MHC allele HLA-A2 has sufficient immunogenic epitopes for the computational work. In the near future, more immunogenic epitopes will be available, and the computational experiments would be conducted on other MHC alleles. The source codes and datasets are available in supporting information files (S1 File).

## Supporting Information

S1 File The source codes and datasets for the GA-based ensemble method. (ZIP)

## References

[1] Nussbaum AK, Kuttler C, Hadeler KP, Rammensee HG, Schild H. PAProC: a prediction algorithm for proteasomal cleavages available on the WWW. Immunogenetics. 2001; 53(2): 87–94. 11345595 10.1007/s002510100300

[2] Kesmir C, Nussbaum A, Schild H, Detours V, Brunak S. Prediction of proteasome cleavage motifs by neural networks. Protein Eng. 2002; 15(4): 287–296. 11983929 10.1093/protein/15.4.287

[3] Bhasin M, Raghava GP. Pcleavage: an SVM based method for prediction of constitutive proteasome and immunoproteasome cleavage sites in antigenic sequences. Nucleic Acids Res. 2005; 33:W202–207. 15988831 10.1093/nar/gki587 PMC1160263

[4] Bhasin M, Raghava GP. Analysis and prediction of affinity of TAP binding peptides using cascade SVM. Protein Sci. 2004; 13(3):596–607. 14978300 10.1110/ps.03373104 PMC2286721

[5] Peters B, Bulik S, Tampe R, Van Endert PM, Holzhutter HG. Identifying MHC class I epitopes by predicting the TAP transport efficiency of epitope precursors. J Immunol. 2003; 171(4):1741–1749. 12902473 10.4049/jimmunol.171.4.1741

[6] Parker KC, Bednarek MA, Coligan JE. Scheme for ranking potential HLA-A2 binding peptides based on independent binding of individual peptide side-chains. Journal of Immunology. 1994; 152(1):163–175. 8254189

[7] Donnes P, Elofsson A. Prediction of MHC class I binding peptides, using SVMHC. BMC Bioinformatics. 2002; 3: 25 12225620 10.1186/1471-2105-3-25 PMC129981

[8] Nielsen M, Lundegaard C, Worning P, Hvid CS, Lamberth K, Buus S, et al Improved prediction of MHC class I and class II epitopes using a novel Gibbs sampling approach. Bioinformatics. 2004; 20(9):1388–1397. 14962912 10.1093/bioinformatics/bth100

[9] Reche PA, Glutting JP, Zhang H, Reinherz EL. Enhancement to the RANKPEP resource for the prediction of peptide binding to MHC molecules using profile. Immunogenetics. 2004; 56(6):405–419. 15349703 10.1007/s00251-004-0709-7

[10] Antes I, Siu SW, Lengauer T. DynaPred: a structure and sequence based method for the prediction of MHC class I binding peptide sequences and conformations. Bioinformatics. 2006; 22(14):e16–24. 16873467 10.1093/bioinformatics/btl216

[11] Donnes P, Kohlbacher O. SVMHC: a server for prediction of MHC-binding peptides. Nucleic Acids Res. 2006; 34: W194–197. 16844990 10.1093/nar/gkl284 PMC1538857

[12] Wan J, Liu W, Xu Q, Ren Y, Flower DR, Li T. SVRMHC prediction server for MHC-binding peptides. BMC Bioinformatics. 2006; 7:463 17059589 10.1186/1471-2105-7-463 PMC1626489

[13] Liu W, Meng X, Xu Q, Flower DR, Li T. Quantitative prediction of mouse class I MHC peptide binding affinity using support vector machine regression (SVR) models. BMC Bioinformatics. 2006; 7:182 16579851 10.1186/1471-2105-7-182 PMC1513606

[14] Liu W, Wan J, Meng X, Flower DR, Li T. In silico prediction of peptide-MHC binding affinity using SVRMHC. Methods Mol Biol. 2007; 409:283–291. 10.1007/978-1-60327-118-9_20 18450008

[15] Nielsen M, Lundegaard C, Blicher T, Lamberth K, Harndahl M, Justesen S, et al NetMHCpan, a method for quantitative predictions of peptide binding to any HLA-A and-B locus protein of known sequence. PLOS One. 2007; 2(8): e796 17726526 10.1371/journal.pone.0000796 PMC1949492

[16] Reche PA, Reinherz EL. Prediction of peptide-MHC binding using profiles. Methods Mol Biol.2007; 409:185–200. 10.1007/978-1-60327-118-9_13 18450001

[17] Jacob L, Vert JP. Efficient peptide-MHC-I binding prediction for alleles with few known binders. Bioinformatics. 2008; 24(3):358–366. 18083718 10.1093/bioinformatics/btm611

[18] Hakenberg J, Nussbaum AK, Schild H, Rammensee HG, Kuttler C, Holzhutter HG, et al MAPPP: MHC class I antigenic peptide processing prediction. Appl Bioinformatics. 2003; 2(3):155–158. 15130801

[19] Tenzer S, Peters B, Bulik S, Schoor O, Lemmel C, Schatz MM, et al Modeling the MHC class I pathway by combining predictions of proteasomal cleavage, TAP transport and MHC class I binding. Cell Mol Life Sci. 2005; 62(9):1025–1037. 15868101 10.1007/s00018-005-4528-2 PMC11924537

[20] Donnes P, Kohlbacher O. Integrated modeling of the major events in the MHC class I antigen processing pathway. Protein Science. 2005; 14(8):2132–2140. 15987883 10.1110/ps.051352405 PMC2279325

[21] Larsen MV, Lundegaard C, Lamberth K, Buus S, Brunak S, Lund O, et al An integrative approach to CTL epitope prediction: a combined algorithm integrating MHC class I binding, TAP transport efficiency, and proteasomal cleavage predictions. Eur J Immunol. 2005; 35(8):2295–2303. 15997466 10.1002/eji.200425811

[22] Rudolph MG, Luz JG, Wilson IA. Structural and thermodynamic correlates of T cell signaling. Annu Rev Biophys Biomol Struct. 2002; 31:121–149. 11988465 10.1146/annurev.biophys.31.082901.134423

[23] Silver ML, Guo HC, Strominger JL, Wiley DC. Atomic structure of a human MHC molecule presenting an influenza virus peptide. Nature. 1992; 360(6402):367–369. 1448154 10.1038/360367a0

[24] Stewart-Jones GB, McMichael AJ, Bell JI, Stuart DI, Jones EY. A structural basis for immunodominant human T cell receptor recognition. Nat Immunol.2003; 4(7):657–663. 12796775 10.1038/ni942

[25] Tung CW and Ho SY. POPI: predicting immunogenicity of MHC class I binding peptides by mining informative physicochemical properties. Bioinformatics. 2007; 23:942–949. 17384427 10.1093/bioinformatics/btm061

[26] Tung CW, Ziehm M, Kämper A, Kohlbacher O, Ho SY. POPISK: T-cell reactivity prediction using support vector machines and string kernels. BMC Bioinformatics. 2011; 12: 446 10.1186/1471-2105-12-446 22085524 PMC3228774

[27] Saethang T, Hirose O, Kimkong I, Tran VA, Dang XT, Nguyen LA, et al PAAQD: Predicting immunogenicity of MHC class I binding peptides using amino acid pairwise contact potentials and quantum topological molecular similarity descriptors. Journal of Immunological Methods. 2013; 387:293–302. 10.1016/j.jim.2012.09.016 23058674

[28] Zhang W, Liu J, Xiong Y, Ke M, Zhang K. Predicting immunogenic T-cell epitopes by combining various sequence-derived features. 2013 IEEE international conference on Bioinformatics and Biomedicine (BIBM). 2013, pp. 4–9, December 18–21; Shanghai.

[29] Rammensee H, Bachmann J, Emmerich NP, Bachor OA, Stevanović S. SYFPEITHI: database for MHC ligands and peptide motifs. Immunogenetics. 1999; 50:213–219 10602881 10.1007/s002510050595

[30] Vita R, Zarebski L, Greenbaum JA, Emami H, Hoof I, Salimi N, et al The immune epitope database 2.0. Nucleic Acids Res.2010; 38: D854–62. 10.1093/nar/gkp1004 19906713 PMC2808938

[31] Kawashima S, Pokarowski P, Pokarowska M, Kolinski A, Katayama T, Kanehisa M. AAindex: amino acid index database, progress report. Nucleic Acids Res. 2008; 36: D202–D205. 17998252 10.1093/nar/gkm998 PMC2238890

[32] Park KJ, Kanehisa M. Prediction of protein subcellular locations by support vector machines using compositions of amino acids and amino acid pairs. Bioinformatics. 2003; 19(13):1656–1663. 12967962 10.1093/bioinformatics/btg222

[33] Chen J, Liu H, Yang J, Chou K. Prediction of linear B-cell epitopes using amino acid pair antigenicity scale. Amino Acids. 2007; 33(3): 423–428. 17252308 10.1007/s00726-006-0485-9

[34] Zhang W, Liu J, Xiong Y. Prediction of conformational B-cell epitopes from 3D structures by random forest with a distance-based feature. BMC Bioinformatics. 2011; 12: 341 10.1186/1471-2105-12-341 21846404 PMC3228550

[35] Li L, William SN. Combining Pairwise Sequence Similarity and Support Vector Machines for Detecting Remote Protein Evolutionary and Structural Relationships. Journal of Computational Biology. 2003; 10(6): 857–868. 14980014 10.1089/106652703322756113

[36] Dubchak I, Muchnik I, Holbrook SR, Kim S-H. Prediction of protein folding class using global description of amino acid sequence. Proc Natl Acad Sci. 1995; 92(19):8700–8704. 7568000 10.1073/pnas.92.19.8700 PMC41034

[37] Horne DS. Prediction of protein helix content from an autocorrelation analysis of sequence hydrophobicities. Biopolymers. 1988, 27(3):451–477. 3359010 10.1002/bip.360270308

[38] Chou KC. Prediction of protein subcellular locations by incorporating quasi-sequence-order effect. Biochemical and biophysical research communications. 2000; 278(2):477–483. 11097861 10.1006/bbrc.2000.3815

[39] Chou KC. Prediction of protein cellular attributes using pseudo-amino acid composition. Proteins: Structure, Function, and Bioinformatics. 2001, 43(3):246–255. 11288174 10.1002/prot.1035

[40] Chou KC. Using amphiphilic pseudo amino acid composition to predict enzyme subfamily classes. Bioinformatics. 2005, 21(1):10–19. 15308540 10.1093/bioinformatics/bth466

[41] Adamczak R, Porollo A, Meller J. Combining prediction of secondary structure and solvent accessibility in proteins. Proteins. 2005; 59(3):467–475. 15768403 10.1002/prot.20441

[42] Zhang W, Niu Y, Xiong Y, Yu W, Zhao M, Liu J. Computational prediction of conformational B-cell epitopes from antigen primary structures by ensemble learning. PLOS One. 2012; 7(8): e43575 10.1371/journal.pone.0043575 22927994 PMC3424238

[43] Abeel T, Helleputte T, Van Y, Dupont P, Saeys Y. Robust biomarker identification for cancer diagnosis with ensemble feature selection methods. Bioinformatics. 2010; 26(3):392–398. 10.1093/bioinformatics/btp630 19942583

[44] Breiman L. Random Forests. Mach. Learn. 2001; 45:5–32.

